# CRISPR/Cas9-Mediated Mutagenesis of *MdCNGC2* in Apple Callus and VIGS-Mediated Silencing of *MdCNGC2* in Fruits Improve Resistance to *Botryosphaeria dothidea*

**DOI:** 10.3389/fpls.2020.575477

**Published:** 2020-11-09

**Authors:** Huijuan Zhou, Suhua Bai, Nan Wang, Xiaohong Sun, Yugang Zhang, Jun Zhu, Chaohua Dong

**Affiliations:** ^1^Key Laboratory of Plant Biotechnology of Shandong Province, College of Life Sciences, Qingdao Agricultural University, Qingdao, China; ^2^Qingdao Key Laboratory of Genetic Improvement and Breeding in Horticultural Plants, Qingdao Agricultural University, Qingdao, China; ^3^Shandong Province Key Laboratory of Applied Mycology, Qingdao, China; ^4^College of Horticulture, Qingdao Agricultural University, Qingdao, China

**Keywords:** *Botryosphaeria dothidea*, gene editing, immune defense, *Malus domestica*, apple callus

## Abstract

Cyclic nucleotide-gated ion channels (CNGCs) have been reported to be involved in multiple plant physiological processes. Their involvement in plant immunity has been studied in several herbal plant species. It remains unclear whether CNGCs in woody plants play a similar role in plant immunity. In the present study, we identified an apple CNGC (designated as MdCNGC2), which is the homolog of *Arabidopsis* CNGC2. Analysis of tissue distribution revealed that *MdCNGC2* was expressed in all tested tissues. Abundant transcripts of *MdCNGC2* were observed in leaves and shoot bark. Low expression was observed in fruits and roots. *MdCNGC2* expression was induced in apple callus and shoot bark by *Botryosphaeria dothidea*. The induction of *MdCNGC2* was significantly higher in susceptible cultivars “Fuji,” “Ralls Janet,” and “Gala” compared to the resistant cultivar “Jiguan,” suggesting that MdCNGC2 may be a negative regulator of resistance to *B. dothidea*. *MdCNGC2* mutagenesis mediated by gene editing based on the CRISPR/Cas9 system led to constitutive accumulation of SA in apple callus. A culture filtrate of *B. dothidea* (BCF) induced the expression of several defense-related genes including *MdPR1*, *MdPR2*, *MdPR4*, *MdPR5*, *MdPR8*, and *MdPR10a*. Moreover, the induction of these genes was significantly higher in *mdcngc2* mutant (MUT) callus than in wild type (WT) callus. Further analysis showed that the spread of *B. dothidea* was significantly lower on MUT callus than on WT callus. Knockdown of the *MdCNGC2* gene reduced lesions caused by *B. dothidea* in apple fruits. These results collectively indicate that *MdCNGC2* is a negative regulator of resistance to *B. dothidea* in apple callus.

## Introduction

During infection by pathogenic microorganisms, a change in the intracellular ion flux is one of the earliest responses of plant cells (Moeder et al., 2011). In particular, Ca^2+^ is thought to play a crucial role in the transduction of infection signals and the initiation of defense responses against pathogen infection (Yang and Poovaiah, 2003). Pathogen infection causes fluctuations in Ca^2+^ flux and then leads to immune responses warding off invaded pathogens. In this process, the calcium channels play crucial roles. The cyclic nucleotide-gated ion channel (CNGC) is a non-selective cation channel that is involved in the regulation of Ca^2+^ influx and defense responses to pathogen infection (Kaplan et al., 2007; Zelman et al., 2012).

The first plant CNGC was reported by Schuurink et al. (1998) in the process identifying the calmodulin binding transporter (HvCBT1) in barley. The structure of plant CNGC protein is similar to that of animal CNGC protein, which contains a transmembrane domain composed of six α-helices (S1–S6) and a pore for ion conduction consisting of 20–30 amino acid residues between S5 and S6. In the C-terminal, there is a cyclic nucleotide-binding domain comprising three α-helices and two β-sheets (Zagotta and Siegelbaum, 1996). At present, CNGCs have been identified in many plant species, such as Arabidopsis (Ma et al., 2009), rice (Nawaz et al., 2014), tomato (Saand et al., 2015), and cabbage (Li et al., 2019). Plant CNGCs play important roles in plant development, the maintenance of intracellular Ca^2+^ homeostasis, and immune defense against pathogenic microorganisms (Duszyn et al., 2019). At least four Arabidopsis CNGCs have been reported to be crucial for defense responses to pathogen infection, i.e., AtCNGC2 (Ali et al., 2007), AtCNGC4 (Chin et al., 2013), AtCNGC11, and AtCNGC12 (Yoshioka et al., 2001, 2006). *AtCNGC2* and *AtCNGC4* mutants (*dnd1* and *dnd2*) demonstrated enhanced broad-spectrum resistance to bacterial pathogens (Clough et al., 2000; Jurkowski et al., 2004; Chin et al., 2013). *AtCNGC2* mutation did not impair gene-for-gene resistance, whereas *AtCNGC4* mutation did impair gene-for-gene resistance (Jurkowski et al., 2004; Chin et al., 2013). Down-regulation of the *AtCNGC2* ortholog in potato and tomato improved resistance to late blight and powdery mildew and resulted in dwarfing and necrosis in tomato but not in potato (Sun et al., 2016). The resistance resulting from down-regulation of the *AtCNGC2* ortholog in tomato and potato was specific to certain pathogens. The double mutation of *AtCNGC11* and *AtCNC12* (cpr22) formed a new chimera, *AtCNGC11/12*, which conferred increased resistance to *Peronospora parasitica* Emco5 (Yoshioka et al., 2006). Recently, a rice CNGC was reported to play a crucial role in blast resistance (Wang et al., 2019). All these studies indicate that CNGCs are important for plant disease resistance.

Apple is one of most widely cultivated fruit trees in China. In recent years, apple trees and fruits have frequently been infected by *Botryosphaeria dothidea*, a causal agent of ring rot. *B. dothidea* causes warts on the bark of stem and shoots, which finally lead to the death of apple trees. In the fruit, *B. dothidea* infection results in brown-ringed necrotic spots and further leads to fruit rot. In comparison, resistant apple cultivars show reduced lesions on fruits and a longer incubation period or lower wart density in shoots or stems (Yan et al., 2005; Tang et al., 2012; Zhang et al., 2018). To control apple ring rot disease, it is necessary to investigate the defense mechanisms of the host plant. Some genes associated with host resistance to *B. dothidea* have been reported. It was found that resistance to *B. dothidea* is affected by E3 ubiquitin ligases, ethylene-response factor, and the salicylic acid signaling pathway (He et al., 2018; Han et al., 2019; Zhao et al., 2019). Although the CNGC family has been well-studied in several model plants, including Arabidopsis, rice, and tomato, apple CNGCs have seldom been reported. In our previous study, we reported that an apple CNGC gene, *MdCNGC1*, is associated with the immune defense of apple plants (Zhang et al., 2018). Its ectopic overexpression enhanced the susceptibility of *Nicotiana benthamiana* to bacterial and fungal pathogens. To further explore the defense mechanism of apple plants with respect to *B. dothidea*, in the present study, we cloned another apple CNGC gene, the homolog of *AtCNGC2*, and designated this as *MdCNGC2*. Since it is difficult to obtain transgenic apple plants, we first performed gene editing of *MdCNGC2* in apple “orin” callus to test its function in immune defense against *B. dothidea*. We found that *MdCNGC2* mutation resulted in strengthened immune responses and improved resistance of apple callus to *B. dothidea*.

## Materials and Methods

### Plant Materials

Apple cultivars “Fuji,” “Ralls Janet,” “Gala,” and “Jiguan” were grown in a greenhouse with natural light and temperature conditions. “Fuji,” “Ralls Janet,” and “Gala” are susceptible to apple ring rot disease, and “Jiguan” shows significant resistance to *B. dothidea* (Yan et al., 2005). The leaves of “Fuji” were used for cDNA synthesis and gene cloning. Current-year shoots and fruits were collected from “Fuji” apple trees, and leaves and roots were collected from “Fuji” apple plantlets. The four tissues or organs were used for gene expression analysis in different tissues. For induced expression, calli were collected 72 h after inoculation with *B. dothidea*. Non-inoculated calli were used as a control. Shoot bark was collected from current-year shoots when the warts just appeared. Mock-infected shoot bark was used as a control.

### Pathogen Inoculation and Preparation of the Culture Filtrate

*Botryosphaeria dothidea* was cultured on PDA plates. Inoculation of current-year shoots was performed according to a previously reported method (Zhang et al., 2018). Inoculation of apple callus was carried out using previously described methods (He et al., 2018; Zhao et al., 2019).

A culture filtrate of *B. dothidea* (BCF) was prepared according to a previously reported method (Portal et al., 2018) with slight modification. *B. dothidea* mycelium was inoculated into 250-ml Erlenmeyer flasks containing 100 ml of potato dextrose broth (PDB) and cultured at 25°C under dark conditions with shaking at 150 rpm. The cultures were harvested from five flasks 7 days post inoculation and pooled to form a composite sample. The culture was filtered through gauzes and centrifuged at 4°C and 12,000 × *g* for 30 min to remove mycelia. The resultant supernatant was passed through a membrane filter (0.22 μm; Millipore, United States). The filtrate was diluted fivefold with sterile distilled water and used to treat apple callus for the induction of an immune responses.

### Quantitative Analysis of Gene Expression

Gene expression was analyzed using quantitative real-time PCR (qRT-PCR). RNA extraction and cDNA synthesis were performed with an EasySpin plant RNA rapid extraction kit (Yuanpinghao, China) and PrimeScript^TM^ II 1st Strand cDNA Synthesis Kit (TaKaRa, China), respectively, according to the manuals provided by the manufacturers. QRT-PCR was performed on a Roche 480 II system with ChamQ SYBR Color qPCR Master Mix (Vazyme, China) using the procedure provided by the manufacturer. The *MdEF1-*α gene (XM_008367439.3) was used as an internal reference to normalize the gene expression. The relative gene expression was calculated according to the 2^–ΔCt^ method (Livak and Schmittgen, 2001).

### Gene Editing Based on CRISPR/Cas9 and Genetic Complementation

The *MdCNGC2* gene mutation was generated using gene editing based on the CRISPR/Cas9 system. The editing vector was constructed based on pHDE-35S-Cas9-mCherry-UBQ, which was a gift from Professor Yunde Zhao (Addgene plasmid #78932^1^; RRID: Addgene_78932) (Gao et al., 2016). The MdU6 promoter (Accession No. MT584802, Supplementary Figure S1) was amplified from apple DNA using primers MdU6-F and MdU6-R (Supplementary Table S1) and was used to promote gRNA expression. The *HygR* gene was replaced with the *KanR* gene for the convenience of selection using kanamycin. Target sequences were selected using CCTOP (CRISPR/Cas9 target online predictor) (Stemmer et al., 2015). *Agrobacterium*-mediated transformation was performed using the *Agrobacterium tumefaciens* EHA105 strain. The gene editing vector was introduced into EHA105, which was then suspended in suspension buffer (2.2 g L^–1^ MS, 25 g L^–1^ sucrose, and 200 μM acetosyringone). The bacterial suspension was used to infect the “orin” callus for 15 min. The infected callus was cultured on M1 medium for 2 days and then transferred to selected medium M2 (4.4 g L^–1^ MS, 30 g L^–1^ sucrose, 0.225 mg L^–1^ 6-BA, 1 mg L^–1^ 2,4-D, and 100 mg L^–1^ kanamycin, pH 5.8) and incubated at 25°C for 35 days. The resistant callus was subcultured every 3 weeks.

To examine gene mutation, DNA was extracted from resistance callus, and the target gene was amplified using a PCR program. The PCR product was cloned into the pMD19-T simple vector and sequenced. For each antibiotic-resistant callus line, 20 clones were randomly picked and sent for sequencing. The MdCNGC2 protein was evaluated using immunoblotting. The total protein of apple callus was exacted using an extraction reagent for plant protein (Huaxingbio, China) following the manufacturer’s manual. Immunoblotting was performed with the MdCNGC2-specific antibody raised by Sangon (Shanghai, China).

Genetic complementation was performed by overexpressing the *MdCNGC2* gene in mutant callus. To prevent destruction of the *MdCNGC2* gene by the CRISPR/Cas9 system, silent mutation at PAM sites and target sequences were engineered into the cDNA sequence of rescue plasmid according to a previously reported method (Kimberland et al., 2018).

### Expression Analysis of Pathogenesis-Related (PR) Genes and Measurement of Phytohormones

Apple callus grown for 2 weeks was treated with fivefold diluted BCF. Callus samples were collected at 3 h and 24 h after treatment for analysis of PR gene expression and the measurement of phytohormones. The expression of PR genes was analyzed using qRT-PCR, and the contents of SA, JA, MeSA, and MeJA were measured as described previously (Zhang et al., 2018). All analysis was performed with three positive lines of gene editing.

### Virus-Induced Gene Silencing (VIGS)

VIGS was performed according to previously reported methods (Lv et al., 2019; Zhao et al., 2019) with some modification. Briefly, a cDNA fragment (304 bp) of *MdCNGC2* was inserted into pTRV2 and introduced into the *Agrobacterium* EHA105 strain. Then, the *Agrobacterium* containing pTRV2-*MdCNGC2* was mixed equally with *Agrobacterium* harboring pTRV1. The mixed bacteria were resuspended using infiltration buffer (10 mM MgCl_2_, 200 mM acetosyringone, 10 mM MES, and 0.01% Silwet L-77, pH 5.6) and used for VIGS. “Fuji” apple fruits at 155 DAFB were used for VIGS analysis. After surface sterilization with 75% ethanol, the fruits were injected with the aforementioned agrobacterial mixture, and the fruits were collected at 160 DAFB. Then, the fruits were maintained at 25°C under dark conditions with 90% humidity. For the negative control experiment, the same procedure was conducted with the pTRV2 empty vector as a substitute for pTRV2-*MdCNGC2*. *MdCNGC2* expression was evaluated 7 days after injection using qRT-PCR. For *B. dothidea* inoculation, a small hole (0.5 cm) was made with a hole puncher followed by inoculation the same size as the mycelium plug. The inoculated fruits were incubated at 25°C under dark conditions with 90% humidity.

## Results

### Cloning and Characterization of *MdCNGC2*

**FIGURE 1:**
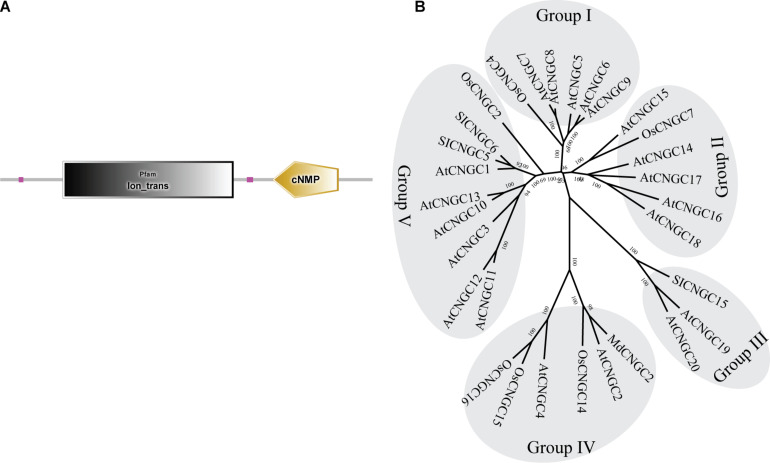
Structural and phylogenetic analyses of the MdCNGC2 protein. **(A)** Domain composition of the MdCNGC2 protein was analyzed using the SMART online program. **(B)** The phylogenetic tree was constructed with CNGC proteins (Table 2) from Arabidopsis, rice, tomato, and apple using MEGA 7.0 based on the neighbor-joining method (Bootstrap value = 1000).

To determine whether the AtCNGC2 homolog in apple plays a role in the immune responses against *B. dothidea*, we searched the apple genome database and identified a gene with ID MD17G1056400. Full-length cDNA was subsequently amplified from cDNA of apple leaves. Sequencing revealed that the nucleoside sequence of the cloned cDNA is exactly the same as that of MD17G1056400 (designated as *MdCNGC2*). *MdCNGC2* contains 2139-bp nucleotides, encoding a protein of 712 amino acids. The putative molecular weight and isoelectric point of the MdCNGC2 protein are 81.476 KD and 9.58, respectively. Domain analysis showed that the MdCNGC2 protein contains an ion-trans domain and a cyclic nucleotide binding domain (Figure 1A). The ion-trans domain is often found in sodium, potassium, and calcium ion channel proteins. It has six transmembrane helices, of which the last two flank a loop that determines ion selectivity. A phylogenetic tree was constructed using CNGC proteins (Supplementary Table S2) from Arabidopsis, rice, tomato, and apple (Figure 1B). These proteins were divided into five groups, and MdCNGC2 clustered with AtCNGC2, AtCNGC4, OsCNGC14, OsCNGC15, and OsCNGC16; most of these proteins are reported to be associated with plant disease resistance (Ali et al., 2007; Chin et al., 2013; Nawaz et al., 2014). We speculated that MdCNGC2 might also be involved in the defense responses of apple plants against pathogen infection.

### Tissue Distribution of *MdCNGC2* Expression and Its Response to *B. dothidea* Infection

**FIGURE 2:**
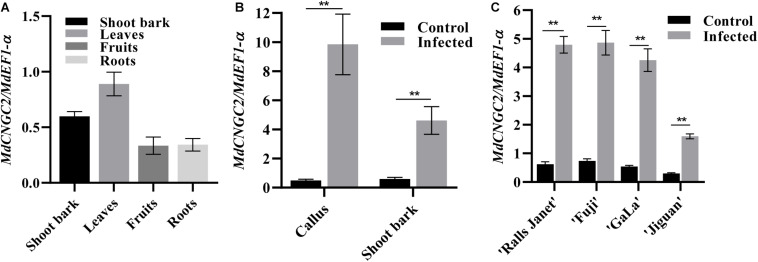
*MdCNGC2* expression in different tissues or different cultivars. **(A)** *MdCNGC2* expression in different tissues. **(B)** *MdCNGC2* expression was induced by *B. dothidea* in callus and shoot bark. Calli were collected 72 h after inoculation wit *B. dothidea*. Non-inoculated calli were used as a control. Shoot bark of “Fuji” was collected when the warts had just appeared. Mock-infected shoot bark was used as a control. **(C)** *MdCNGC2* expression in the shoot bark of four cultivars with different levels of resistance to *B. dothidea*. Statistical significance was determined using one-way ANOVA followed by Tukey’s test **(A)** or two-way ANOVA followed by Bonferroni’s test **(B,C)**. Data were derived from four biological replicates and are presented as means ± SD. Double asterisks indicate *p* < 0.01.

*MdCNGC2* expression in shoot bark, fruits, leaves, and roots was examined using qRT-PCR. The results showed that *MdCNGC2* expression could be detected in all tested tissues of apple plants but exhibited different expression levels. Higher levels of *MdCNGC2* expression were observed in leaves and shoot bark (Figure 2A). In contrast, fruits and roots exhibited lower *MdCNGC2* expression. To determine whether *MdCNGC2* expression was affected by *B. dothidea*, we inoculated “orin” callus and current-year shoots of “Fuji” with *B. dothidea* and analyzed the *MdCNGC2* expression in inoculated tissues. The calli used for gene expression analysis were collected 72 h after inoculation. We found that *MdCNGC2* expression in calli was significantly (*p* < 0.01) enhanced 72 h after inoculation compared with the control (Figure 2B). Due to the long incubation period (1 month) of shoot bark after inoculation with *B. dothidea*, only the shoots on which the symptoms (warts) had just appeared were used for expression analysis. In inoculated shoot bark, *MdCNGC2* expression also significantly increased compared with the mock-infected control. However, the enhancement of *MdCNGC2* expression in inoculated shoot bark was lower than that in inoculated “orin” callus (Figure 2B). *MdCNGC2* expression was also compared among resistant and susceptible cultivars. “Jiguan” is highly resistant to *B. dothidea*, whereas “Fuji,” “Gala,” and “Ralls Janet” are susceptible cultivars (Yan et al., 2005). Susceptible cultivars “Fuji,” “Gala,” and “Ralls Janet” showed higher basal and induced expression than resistant cultivars “Jiguan” and “Gala” (Figure 2C).

### Gene Editing Based on the CRISPR/Cas9 System Blocks *MdCNGC2* Expression in Callus

**FIGURE 3:**
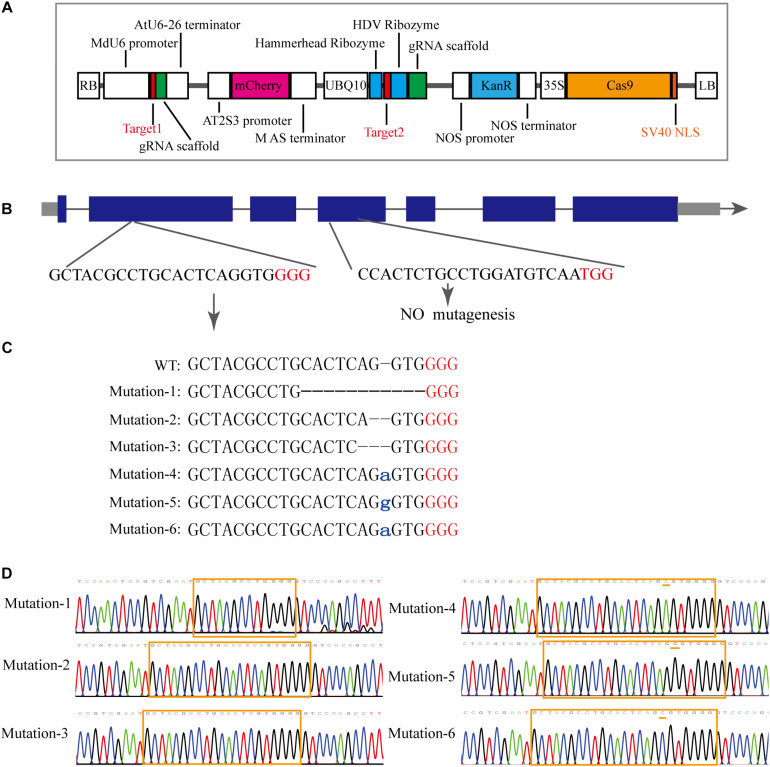
Mutagenesis of the *MdCNGC2* gene based on the CRISPR/Cas9 system. **(A)** Expression cassette of the gene-editing vector derived from pHDE-35S-Cas9-mCherry-UBQ. **(B)** Genomic profile of the *MdCNGC2* gene. Blue rectangles represent exons of the *MdCNGC2* gene. **(C)** *MdCNGC2* mutations resulted from three positive lines of gene editing. WT, wild type. Lowercase letters indicate inserted bases. Red letters indicate PAM sites. **(D)** Sequencing chromatogram of partial genomic DNA of *MdCNGC2* corresponding to the mutations. The orange frame indicates target sites for gene editing, and orange dashes show base insertions.

**FIGURE 4:**
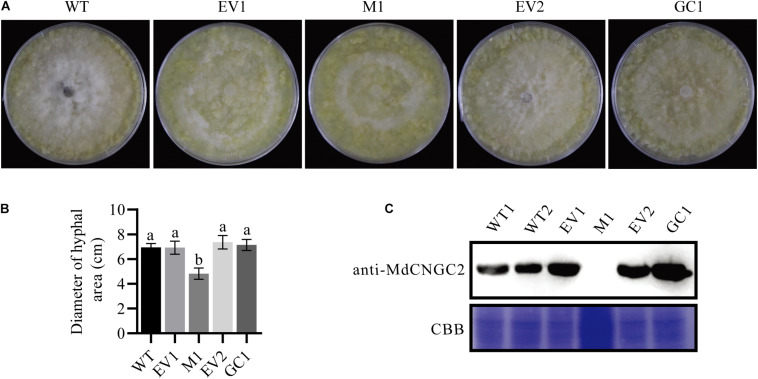
Mutation of *MdCNGC2* inhibits the hyphal spread of *B. dothidea* on apple callus. The experiments were performed with three mutation lines. The representative data are presented, and the data from the two other mutation lines are shown in Supplementary Figure S2. **(A)** Hyphal spread on apple callus at 120 hpi. **(B)** Quantitation of hyphal spread of *B. dothidea* on apple callus. Different letters indicate significant differences (*p* < 0.05). Bars represent the mean ± SD (*n* = 5). Statistical significance was determined using one-way ANOVA followed by Tukey’s test. **(C)** MdCNGC2 protein expressed in different apple calli detected using immunoblotting. EV1, the callus transformed with pHDE-35S-Cas9-mCherry-UBQ, an empty vector used here for gene editing; M1, apple callus with the mutant *MdCNGC2* gene; EV2, the callus transformed with pRI101, an empty vector for genetic complementation. GC1, the callus expressing the *MdCNGC2* gene with silent mutation at the PAM site and target sequences. WT1 and WT2, repeat loading of the protein sample exacted from wild-type callus. CBB, staining by Coomassie brilliant blue shows protein samples with equal loading volumes.

To investigate the *MdCNGC2* function, gene editing based on the CRISPR/Cas9 system was conducted in apple callus for mutagenesis of the *MdCNGC2* gene. We selected the target sequences from exons of the *MdCNGC2* gene using the online program CCTOP. To ensure successful mutagenesis, we selected two target sites (Figure 3B). The target sequences were verified for their specificity to the *MdCNGC2* gene through a BLAST search against the apple genome database. The first target sequence was fused with the MdU6 promoter (Supplementary Figure S1) and gRNA sequence and was integrated into the *Spe*I site of the plasmid vector pHDE-35S-Cas9-mCherry-UBQ, a vector for gene editing used in *Arabidopsis* (Gao et al., 2016). The second sequence was fused with a gRNA sequence and an RGR sequence (Gao and Zhao, 2014), and the fused sequence was then inserted downstream of the UBQ10 promoter. We replaced the *HygR* gene with the *KanR* gene to use kanamycin to screen positive transformants (Figure 3A). The new construct was introduced into “orin” callus using *Agrobacterium*-mediated transformation methods. Kanamycin-resistant callus was subcultured twice. Then, positive calli were subjected to molecular examination. Sequencing showed that the positive callus exhibited high mutant efficiency. We sequenced 10 positive callus lines, and all sequenced calli showed a sequence mutation in the *MdCNGC*2 gene. Each line contained two or three different sequence mutations in the *MdCNGC2* gene, and a total of six different sequence mutations were found in all sequenced calli. The mutations were obtained at the first target site, including base deletions of 10 bp, 1 bp, and 2 bp, as well as insertion of an adenine (A), a guanine (G), or a cytosine (C). Figures 3C,D show all six mutations in the three selected lines. The second target sites did not show any mutation. *MdCNGC2* expression was also examined at the protein level using immunoblotting. *MdCNGC2* expression was completely blocked in mutant callus (Figure 4C).

### *MdCNGC2* Silencing Improves Resistance of Apple Callus and Fruit to *B. dothidea*

To investigate whether *MdCNGC2* mutation affects the growth of pathogenic fungus on callus, we inoculated WT, empty vector-transformed (EV1), and MUT calli with *B. dothidea*. *B. dothidea* growth was significantly reduced on MUT callus compared to WT and EV1 calli (Figures 4A,B and Supplementary Figure S2). There was no significant difference in *B. dothidea* between EV1 and WT calli. To confirm that this phenotype was caused by *MdCNGC2* mutation, we performed genetic complementation (GC) by overexpressing the *MdCNGC2* gene. To prevent destruction by the CRISPR/Cas9 system, the target sequence and PAM sites were replaced with a synonymous codon. The obtained *MdCNGC2*-containing callus exhibited *MdCNGC2* expression comparable to that of the WT and empty vector-transformed (EV2) calli (Figure 4C). *B. dothidea* growth was recovered on the GC callus (Figures 4A,B and Supplementary Figure S2). Enhanced growth was observed in GC callus compared to MUT and EV2, indicating that *MdCNGC2* expression is favorable to *B. dothidea* growth on apple callus.

**FIGURE 5:**
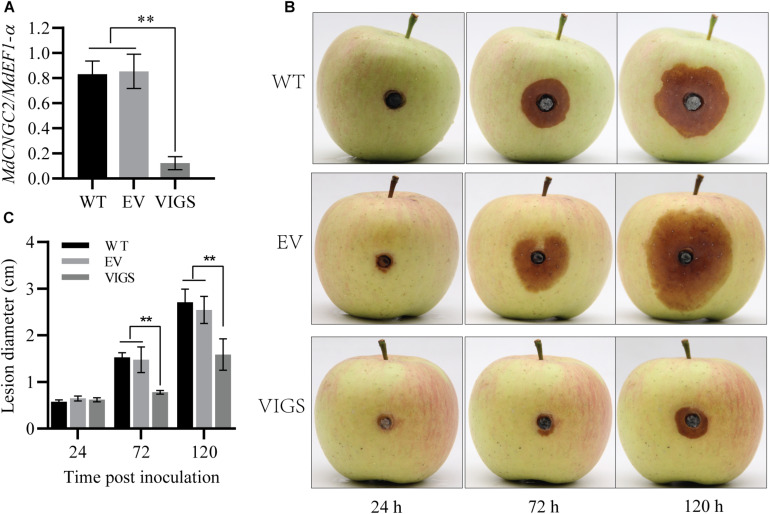
Effects of *MdCNGC2* on the resistance of apple fruits to *B. dothidea*. **(A)** *MdCNGC2* expression was reduced by VIGS. WT, wild type, without any treatment before inoculation. EV, injected with a mixture of agrobacteria containing empty vectors, pTRV1 or pTRV2. VIGS, injected with a mixture of agrobacteria harboring pTRV1 or pTRV2-*MdCNGC2*. The data are presented as the mean ± SD (*n* = 4). Statistical significance was determined by one-way ANOVA followed by Tukey’s *post hoc* test. Asterisks indicate a significant difference (*p* < 0.05). **(B)** Apple fruits were inoculated with *B. dothidea* 7 days after agrobacterial injection. Photographs were taken at 24, 72, and 120 hpi. **(C)** Quantitation of lesions resulting from *B. dothidea* by measuring diameters. The data are presented as the mean ± SD (*n* = 5). Statistical significance was determined by two-way ANOVA followed by Tukey’s *post hoc* test. Asterisks indicate a significant difference (**p < 0.01).

To verify the effect of *MdCNGC2* mutation on the resistance to *B. dothidea*, we knocked down *MdCNGC2* in apple fruits using VIGS as previously reported (Zhao et al., 2019). Then, the fruits were inoculated with *B. dothidea*, and lesions were evaluated at 24, 72, and 120 hpi. *MdCNGC2* expression was reduced by 80% after VIGS (Figure 5A). Reduction of *MdCNGC2* expression led to decreased lesions of fruits resulting from *B. dothidea* (Figures 5B,C), indicating that *MdCNGC2* negatively regulated apple resistance to *B. dothidea.*

### *MdCNGC2* Mutation Affects Immune Responses of Calli to *B. dothidea*

**FIGURE 6:**
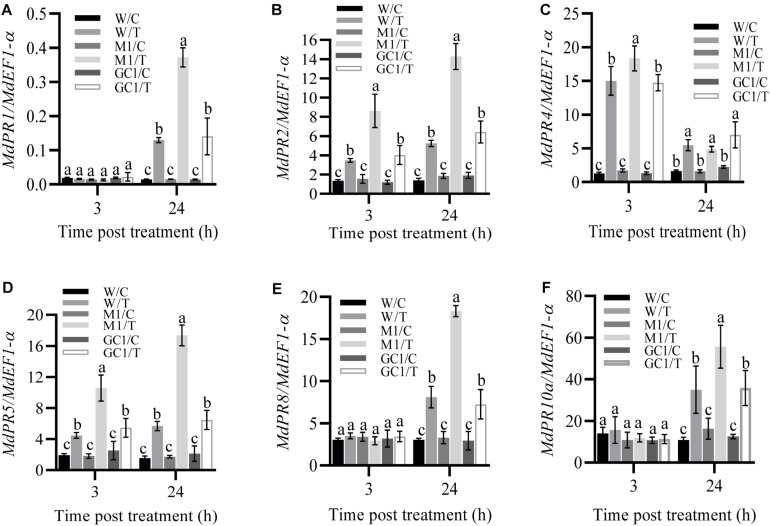
Effects of *MdCNGC2* mutation on the expression of defense-related genes. **(A–F)** show the relative expression of *MdPR1*, *MdPR2*, *MdPR4*, *MdPR5*, *MdPR8* and *MdPR10a*, respectively, in M1 line compared to WT and GC1 lines. W/C, WT callus treated with 5 × diluted PDB and used as a control. W/T, WT callus treated with BCF. M1/C, mutated callus treated with 5 × diluted PDB was used as a control. M1/T, mutated callus treated with BCF. GC1, the callus expressing the *MdCNGC2* gene with silent mutation at the PAM site and target sequences. The expression of pathogenesis-related (PR) genes was determined using qRT-PCR. The *MdEF1-*α gene was used as an internal reference. The experiments were performed with three mutation lines. The representative data are presented (mean ± SD, *n* = 4), and the data from the other two mutation lines are shown in Supplementary Figures S3, S4. Statistical significance was determined by two-way ANOVA followed by Tukey’s *post hoc* test. Different letters indicate a significant difference (*p* < 0.05).

To further investigate the effect of *MdCNGC2* on the immune responses of apple plants, we examined the expression of several immune-related genes, including *MdPR1*, *MdPR2*, *MdPR5*, *MdPR8* (Quaglia et al., 2011), and *MdPR10a* (Pühringer et al., 2000). Apple callus was treated with fivefold diluted BCF, and gene expression was analyzed at 3 h and 24 h after treatment. We found that the expressions of *MdPR1*, *MdPR8*, and *MdPR10a* significantly increased in both wild type (WT) and *mdcngc2* mutant calli (MUT) but only at 24 h after treatment with BCF (Figures 6A,E,F and Supplementary Figures S3, S4). Moreover, the MUT callus exhibited significantly higher expression of these genes than the WT callus. The expressions of three other genes, *MdPR2*, *MdPR4*, and *MdPR5*, were significantly enhanced at both 3 h and 24 h after treatment (Figures 6B–D and Supplementary Figure S4). Moreover, significantly lower expression was also observed in WT calli compared to MUT calli except for *MdPR4. MdPR4* expression was higher in MUT callus than in WT callus at 3 h after treatment with BCF, but no significant difference was observed between WT and MUT calli at 24 h (Figure 6C and Supplementary Figures S3, S4). The expression of all six *PR* genes showed no significant difference between WT and GC calli, indicating that the expression of *MdPR* genes recovered to a level comparable to WT after *MdCNGC2* expression.

**FIGURE 7:**
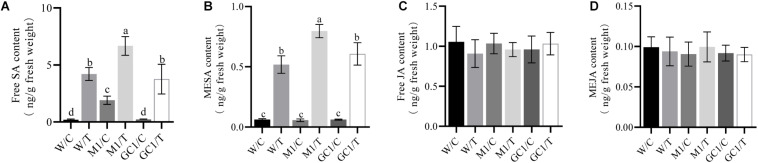
Effects of *MdCNGC2* mutation on SA and JA contents. **(A)** free SA content of orin callus. **(B)** MESA content of orin callus. **(C)** free JA content of orin callus. **(D)** MeJA content of orin callus. W/C, WT treated with 5 × diluted PDB and used as a control. W/T, WT callus treated with BCF. M1/C, mutated calli treated with 5 × diluted PDB were used as a control. M1/T, mutated callus treated with BCF. GC1/C, GC callus treated with 5 × diluted PDB. GC1/T, GC callus treated with BCF. The experiments were performed with three mutation lines. The representative data are presented (mean ± SD, *n* = 5). The data from the other two mutation lines are shown in Supplementary Figure S5. The three mutation callus lines were compared to the same WT control. Statistical significance was determined by one-way ANOVA followed by Tukey’s *post hoc* test. Different letters indicate a significant difference (*p* < 0.05).

We also analyzed the effect of *MdCNGC2* on SA and JA, two important phytohormones related to plant immunity. We found that basal SA levels were significantly different between MUT and WT calli (Figures 7A,B and Supplementary Figures S5a,b,e,f). The basal SA level was significantly higher in MUT callus than in WT callus. Moreover, the SA level was induced by BCF, and the induction level was significantly higher in MUT callus than in WT callus at 24 h after treatment. No significant difference was observed between WT and GC calli. However, no significant difference in basal or induced JA levels was observed between MUT and WT calli (Figures 7C,D and Supplementary Figures S5c,d,g,h).

## Discussion

In this work, we identified and characterized an apple CNGC gene, *MdCNGC2*. *MdCNGC2* is a homolog of Arabidopsis *CNGC2*. CRISPR/Cas9-mediated mutagenesis of *MdCNGC2* increased apple callus resistance to *B. dothidea*, and *MdCNGC2* silencing in fruits also improved the resistance to *B. dothidea*. The present study confirms that *MdCNGC2* has a similar function to *AtCNGC2* in plant immunity and may be used as a potential target gene for improvement of apple resistance to *B. dothidea.* Our results provided a basis for further understanding defense mechanism against *B. dothidea* and controlling ring rot disease.

Analysis of tissue distribution of *MdCNGC2* revealed that *MdCNGC2* was expressed in all tested tissues. This is similar to the *CNGC2* homolog of potato and tomato (Sun et al., 2016) in which *CNGC2* was detected in all test issues. These results collectively suggest that *MdCNGC2* may be a pleiotropic gene, playing a role in different physiological processes. *MdCNGC2* expression could be induced in the target tissue of *B. dothidea*, the shoot bark, and the degree of induction was different among apple cultivars with different levels of resistance to *B. dothidea*. The same phenomenon was also observed in a previous report where *MdCNGC1* expression induced by *B. dothidea* was different in apple cultivars with different levels of resistance to *B. dothidea* (Zhang et al., 2018), suggesting that *MdCNGC2* expression, similar to that of *MdCNGC1*, may be related to *B. dothidea* resistance.

Since it is difficult and time-consuming to obtain transgenic apple plants, we used “orin” callus for further function analysis of *MdCNGC2*. “Orin” callus originated from apple cultivar “orin” and has some traits suitable for experimental study of apple gene function, such as limitless proliferation potential. “Orin” callus can be subcultured without differentiation and is easy to transform using the *Agrobacterium*-mediated method. It has been successfully used in the study of gene function (He et al., 2018; Han et al., 2019; Zhao et al., 2019). To investigate gene function, we established gene deletion technology in “orin” callus based on the CRISPR/Cas9 system. The obtained mutant calli are a mixture of multiple different mutations of *MdCNGC2* genes, not a single mutation as expected. We speculate that the obtained mutant callus is not a clone from a single cell. Although it is not a single-cell clone, immunoblotting assay showed *MdCNGC2* expression was completely blocked in mutant calli. Thus, the mutant callus can be used for investigation of *MdCNGC2* function.

*MdCNGC2* deletion by gene editing led to stronger immune responses of “orin” apple callus to *B. dothidea* and inhibited *B. dothidea* spread on “orin” callus. This indicates that *MdCNGC2* deletion is unfavorable to *B. dothidea* spread on apple callus. This result was supported by VIGS assay in fruits. *MdCNGC2* silencing via VIGS improved fruit resistance to *B. dothidea*. As expected, the results indicate that MdCNGC2 has a similar function to AtCNGC2 (Jurkowski et al., 2004; Ali et al., 2007; Chin et al., 2013) or its homologs of potato and tomato (Sun et al., 2016). In the two species, *CNGC2* silencing enhanced the resistance of plants to oomycete *Phytophthora infestans* and two powdery mildew species, *Oidium neolycopersici* and *Golovinomyces orontii* (Sun et al., 2016).

In other studies, CNGC2 gene deletion resulted in constitutive SA accumulation and enhanced *PR1* expression (Clough et al., 2000; Ma et al., 2009). Here, we also examined the expression of *PR* genes and found that all tested PR genes exhibited a significant induction by BCF, an elicitor that originated from *B. dothidea*. There was no constitutive accumulation of *MdPR1* transcripts, but induced *MdPR1* expression was significantly higher in MUT than in WT callus. Both constitutive and induced SA contents were higher in MUT than in WT callus. Thus, *MdCNGC2* mutation led to an immune response in apple callus partly similar to that in the *dnd1* mutant Arabidopsis. These results collectively indicate that *MdCNGC2* may be used as a target gene to improve the resistance of apple plants to *B. dothidea*.

Although MdCNGC2 mutation improves the resistance of apple callus to *B. dothidea*, other functions of *MdCNGC2* should be considered when *MdCNGC2* is used as a target gene to improve apple resistance to *B. dothidea*. CNGC2 plays multifaceted roles in Arabidopsis, and CNGC2 mutation leads to pleiotropic phenotypes, including dwarf plants and elevated levels of salicylate compounds (Yu et al., 1998; Clough et al., 2000). CNGC2 is also involved in leaf development and senescence programming (Köhler et al., 2001; Ma et al., 2010) as well as fertilization (Rehman, 2014). CNGC2 mutation results in short anthers, poor pollen tube growth, and a fertilization defect. These pleiotropic phenotypes may affect the utility of MdCNGC2 as a target gene for genetic improvement of susceptible apple cultivars.

In summary, *MdCNGC2* is a member of the CNGC family that is involved in the regulation of the immune defense of apple against *B. dothidea*. Its expression is induced by *B. dothidea*, and the induced expression in susceptible cultivars was higher than that in the resistant cultivar. *MdCNGC2* deletion led to constitutive accumulation of SA and *MdPR1* expression. *B. dothidea* spread on MUT callus was inhibited. These results collectively indicate that *MdCNGC2* is a negative regulator of resistance to *B. dothidea* in apple callus.

## Data Availability Statement

The original contributions presented in the study are included in the article/Supplementary Material, further inquiries can be directed to the corresponding author.

## Author Contributions

SB and CD designed the experiments and wrote the manuscript. SB, HZ, NW, CD, and XS performed the experiments. HZ, CD, JZ, and YZ analyzed the data. All authors read and approved the manuscript.

## Conflict of Interest

The authors declare that the research was conducted in the absence of any commercial or financial relationships that could be construed as a potential conflict of interest.

## Footnotes

1 http://n2t.net/addgene:78932
