# Supplementary material for: CRISPR/Cas9-Mediated Mutagenesis of MdCNGC2 in Apple Callus and VIGS-Mediated Silencing of MdCNGC2 in Fruits Improve Resistance to Botryosphaeria dothidea
Source: Front Plant Sci. 2020 Nov 9;11:575477. doi: 10.3389/fpls.2020.575477 (PMC7680757; doi:10.3389/fpls.2020.575477)
Supplement: Supplementary file 1 [file Data_Sheet_1.pdf]

Supplementary Material

1 Supplementary Figures and Tables

1.1 Supplementary Figures

>MdU6 promoter
ACCCGCAAGGAATTTAAGTTAATATGGCCAATTCCTTGATGATGAGAATCATCATT
CCTGTAGCAGTGTTATTAAGAGAATAACCGATGAATTCCTTGCTAACTTATTGGGT
GCTAAGTATTATTGGATCGAGAACATGATTTGTGTTAGTTTTACATATATAAAATA
AATGGGTAAAGAGTTACCTGTTTATAATCACGGTTAATACCTATAACCGCCCATTT
AAATTTTGCGGGTAAACGGTTATACCCATAACCGTTTATTTATCTAAACGGTTATCC
ATAACCGTAACCATTTAATTTAAATGGACGGGTAACCGCGGTTACCCATAACCAAT
GGGTATTTGCCCATCTTTATTTATGATTGAGGATACTCAGTCCACATAGGAAAGCC
CAAGGTGGGAAAAAAAGTTGACCATTAAAAGGACGGAAGCCAGCGTCCCACATCG
GCCAAAACAAGGTTTCTAAACGACTTTATATACTCTTACTTGAAGGTAATGCTT

Figure S1. Sequence of the MdU6 promoter.

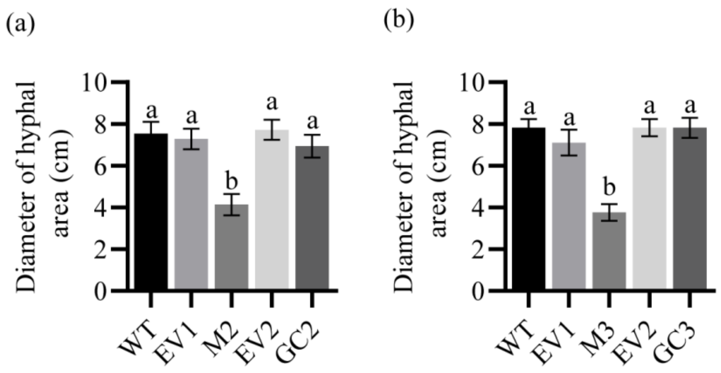

Figure S2. Quantitation of hyphal spread of B. dothidea on mutation callus. (a) The second line and (b) the third line of MdCNGC2 mutation. Different letters indicate a significant difference ($p < 0.05$). Bars represents the mean ± SD (n = 5). Statistical significance was determined using one-way ANOVA followed by Tukey's test. EV1, the callus transformed with pHDE-35S-Cas9-mCherry-UBQ, an empty vector used here for gene editing; M2, M3, apple callus with the mutant MdCNGC2 gene; EV2, the callus transformed with pRI101, an empty vector for genetic complementation. GC2, GC3, the callus expressing the MdCNGC2 gene with silent mutation at the PAM site and target sequences.

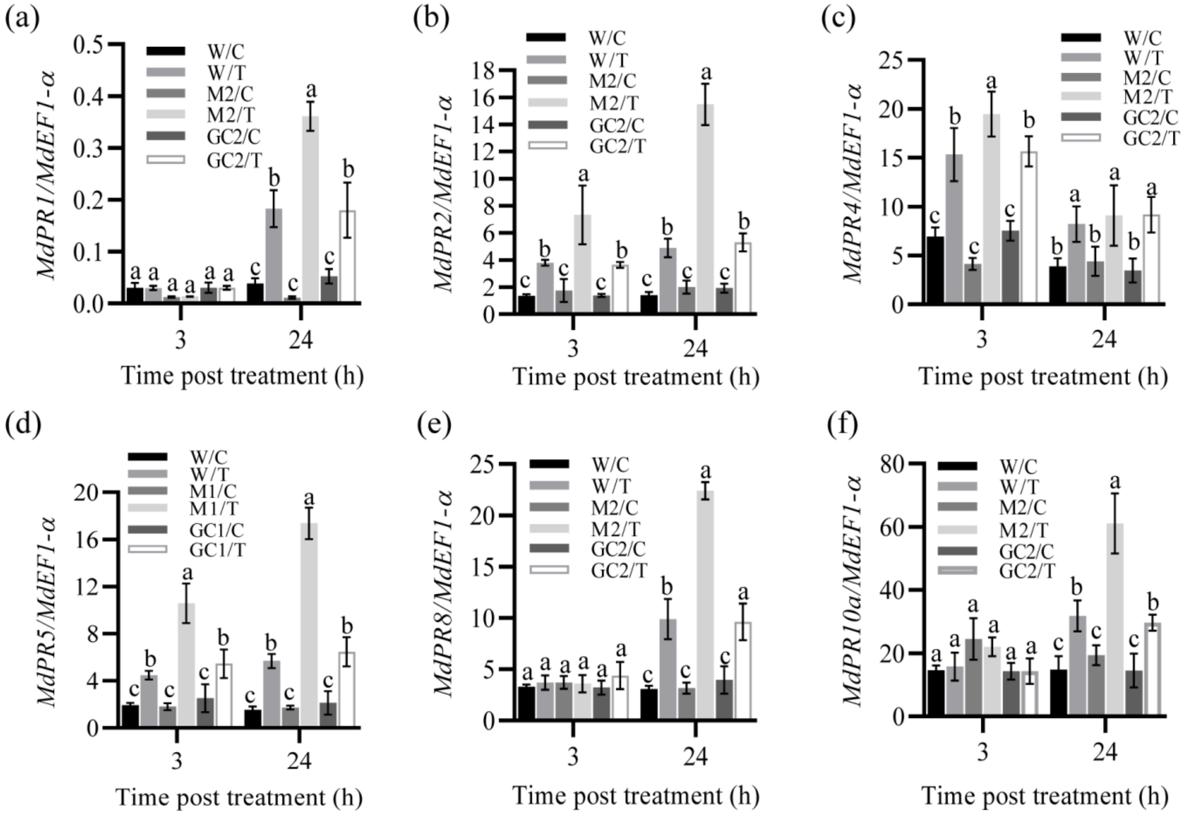

Figure S3. Effects of the second line of *MdCNGC2* mutation on the expression of defense-related genes. a-f show the relative expression of *MdPR1, MdPR2, MdPR4, MdPR5*, *MdPR8* and *MdPR10a*, respectively, in M2 line compared to WT and GC2. W/C, WT callus treated with 5 × diluted PDB and used as a control. W/T, WT callus treated with BCF. M2/C, the second mutated callus line treated with 5 × diluted PDB was used as a control. M2/T, the second mutated callus line treated with BCF. The expression of pathogenesis-related (PR) genes was determined using qRT-PCR. The *MdEF1-α* gene was used as an internal reference. GC2, the callus expressing the *MdCNGC2* gene with silent mutation at the PAM site and target sequences. The data are presented as the mean ± SD (n = 4). Statistical significance was determined by two-way ANOVA followed by Tukey's post-hoc test. Different letters indicate a significant difference ($p < 0.05$).

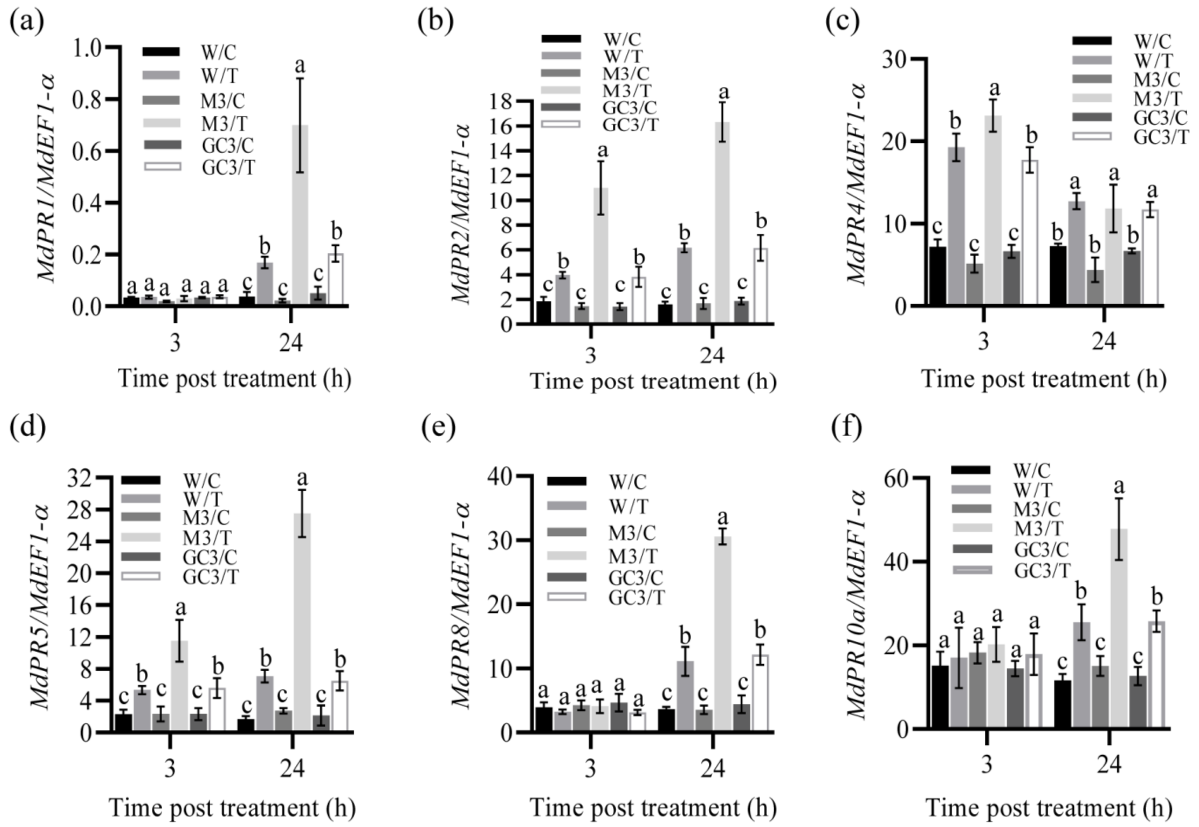

Figure S4. Effects of the third line of *MdCNGC2* mutation on the expression of defense-related genes. a-f show the relative expression of *MdPR1, MdPR2, MdPR4, MdPR5, MdPR8* and *MdPR10a*, respectively, in M3 line compared to WT and GC3. W/C, WT callus treated with 5 × diluted PDB and used as a control. W/T, WT callus treated with BCF. M3/C, the third mutated callus line treated with 5 × diluted PDB was used as a control. M3/T, the third mutated callus line treated with BCF. GC3, the callus expressing the *MdCNGC2* gene with silent mutation at the PAM site and target sequences. The expression of pathogenesis-related (PR) genes was determined using qRT-PCR. The *MdEF1-α* gene was used as an internal reference. The data are presented as the mean ± SD (n = 4). Statistical significance was determined by two-way ANOVA followed by Tukey's post-hoc test. Different letters indicate a significant difference ($p < 0.05$).

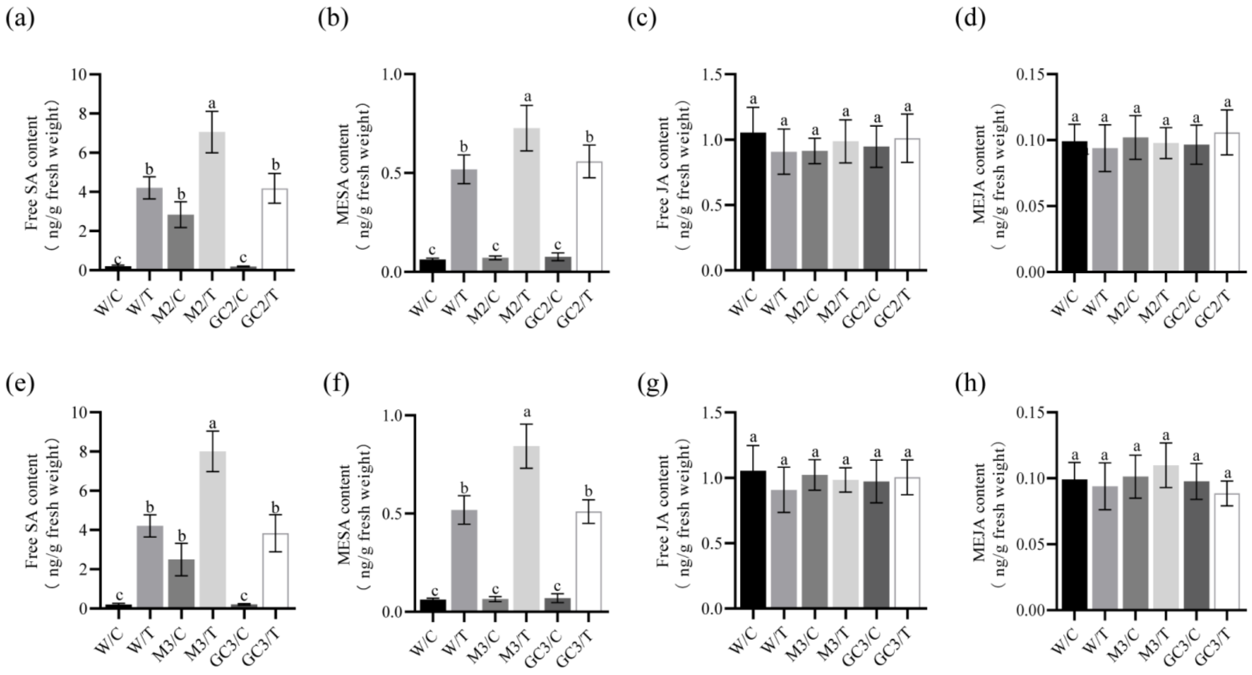

Figure S5. SA and JA contents in the second (a-d) and third (e-h) callus lines of *MdCNGC2* mutation. W/C, WT treated with 5 × diluted PDB and used as a control. The three mutation callus lines were compared to the same WT control. W/T, WT callus treated with BCF. M/C, mutated calli treated with 5 × diluted PDB were used as a control. M/T, mutated callus treated with BCF. GC/C, GC callus treated with 5 × diluted PDB. GC/T, GC callus treated with BCF. The data are presented as the mean ± SD (n = 5). Statistical significance was determined by one-way ANOVA followed by Tukey's post-hoc test. Different letters indicate a significant difference ($p < 0.05$).

1.2 Supplementary Figures and Tables

Table S1. The primers used in this study

Primer name	Primer sequence (5'→3')
**The full-length cDNA primers**	
FL-MdCCNGC2-F	ATGTCCTCCTCCCAGTTCTTCC
FL-MdCNGC2-R	TTATTCAAGGTGATCGTGTGGC
MdU6-F	ACCCGCAAGGAATTTAAGTTAA
MdU6-R	AAGCATTACCTTCAAGTAAGAG
**Primers used for construction of gene editing vector**	
mCNGC2-RG1-F1	CCACTCTGCCTGGATGTCAAGTTTTAGAGCTAGAAATAGC
mCNGC2-RG1-F2	GGACGAAACGAGTAAGCTCGTCCCACTCTGCCTGGATGTCAA
mCNGC2-RG1-F3	GAGTGGCTGATGAGTCCGTGAGGACGAAACGAGTAAGCTCG
MdU6-pHDE-F	GTCAAACACTGATAGTTTAAACCCGCAAGGAATTTAAGTT
MdU6-pHDE-R	CCAAGCTTCACTTCACTAGTCACCTGAGTGCAGGCGTAGC
CNGC2T2-MdU6-R	CACCTGAGTGCAGGCGTAGCAAGCATTACCTTCAAGTAAGAG
CNGC2T2-U6ter-F	GCTACGCCTGCACTCAGGTGGTTTTAGAGCTAGAAATAGC
U6ter-pHDE-R	TTGAGACCAAGCTTCACTTCCCATCAGAGGTGTAACGGAA
RGR-pHDE-F	TTTTTCTGATTAACAGCTCGGAGTGGCTGATGAGTCCGTG
RGR-pHDE-R	GCTAGCTTACTCAGTTAGGTGTCCCATTCGCCATGCCGAA
RGRs-R	GTCCCATTCGCCATGCCGAA
**Primer for detection of mutation in target sequence**	
Crispr-CNGC2-Target1-F	AGAACTCCCAGAGCGACGACA
Crispr-CNGC2-Target1-R	TACGAACGTGAGGCGAGAAAA
Crispr-CNGC2-Target2-F	TCTTGCTCGGACGAAGTTTGC
Crispr-CNGC2-Target2-R	AACGGCGTGGAGAAATACCTG
**Quantitative real-time PCR primers**	
MdCNGC2-192-F	CATCTGCGACATTCACCTGC
MdCNGC2-192-R	ACCTGTAGCGACGCCAAGTA
MdEFa-F	CAAGGCAAGGTACGAGGAA
MdEFa-R	GAAGTGGAAGACGGAGGG
MdPR1-F	AGTAGGCGTTGGTCCCTT
MdPR1-R	ACTGTAGTCGGCTTTCTCC
MdPR2-F	TTGATAATGCGAGGACTT
MdPR2-R	GGGTATTTAGGCTGTTTG
MdPR4-F	CCACATACCACCTCTACAATC
MdPR4-R	AAAGGCAGTCCATCCATAT
MdPR5-F	AACTTGCCTATGTCTGTCGC
MdPR5-R	CCATCAGCCGCTTTCACT
MdPR8-F	CAACTCGGGCAACTACCA
MdPR8-R	GTTCTGATGTCGGCACTCT
MdPR10-F	AAACTACTCATACGCCTACAC
MdPR10-R	TTGATCTCAACATCACCCT
Primers for VIGS	
VIGS-*MdCNGC2*-F	CGGAATTCCCATCTTTTGGGGTTTAA
VIGS-MdCNGC2-R	CGAGCTCTCCATCTCATCTTCTCCC
Primers for genetic complement	
MdCNGC2-mut-F1	GTTATGCATGTACCCAAGTCGGGGTCCCAGCCTTTCACTC
MdCNGC2-mut-R1	GACTTGGGTACATGCATAACATTCGACGGAGTTGGAGATG
MdCNGC2-mut-F2	CCGTTGTGTTTAGACGTAAATGGCACATTTAACTATGGAA

MdCNGC2-mut-R2	TTTACGTCTAAACACAACGGCGGCTTTCTCATCACGCTTG
pRI101-*MdCNGC2-mut*-F	CGGGATCCATGTCCTCCTCCCAGTTCTT
pRI101-*MdCNGC2-mut*-R	ATTCGAGCTCACTAGTGGATCCTCA

Table S2*. The genes used in the present study

Name	Species	Accession
AtCNGC1	*A. thaliana*	At5G53130
AtCNGC2	*A. thaliana*	At5g15410
AtCNGC3	*A. thaliana*	At2g46430
AtCNGC4	*A. thaliana*	At5g54250
AtCNGC5	*A. thaliana*	At5g57940
AtCNGC6	*A. thaliana*	At2g23980
AtCNGC7	*A. thaliana*	At1g15990
AtCNGC8	*A. thaliana*	At1g19780
AtCNGC9	*A. thaliana*	At4g30560
AtCNGC10	*A. thaliana*	At1g01340
AtCNGC11	*A. thaliana*	At2g46440
AtCNGC12	*A. thaliana*	At2g46450
AtCNGC13	*A. thaliana*	At4g01010
AtCNGC14	*A. thaliana*	At2g24610
AtCNGC15	*A. thaliana*	At2g28260
AtCNGC16	*A. thaliana*	At3g48010
AtCNGC17	*A. thaliana*	At4g30360
AtCNGC18	*A. thaliana*	At5g14870
AtCNGC19	*A. thaliana*	At3g17690
AtCNGC20	*A. thaliana*	At3g17700
OsCNGC2	*O. sativa*	Os06g0527100
OsCNGC4	*O. sativa*	ABF97880
OsCNGC7	*O. sativa*	Os02g0627700
OsCNGC14	*O. sativa*	Os03g0758300
OsCNGC15	*O. sativa*	Os01g0782800
OsCNGC16	*O. sativa*	Os05g0502000
MdCNGC2	*Malus domestica*	MD17G1056400
SlCNGC5	*S. lycopersicum*	Solyc06g051920
SlCNGC6	*S. lycopersicum*	Solyc03g007260
MdPR1a	*Malus domestica*	MD05G1109100
MdPR2	*Malus domestica*	MD14G1080100
MdPR4	*Malus domestica*	MD04G1225400
MdPR5	*Malus domestica*	MD09G1256300
MdPR8	*Malus domestica*	MD01G1213300
MdPR10	*Malus domestica*	MD16G1160700

* The gene sequences of apple, Arabidopsis, rice and tomato were retrieved from genomic database of apple (https://iris.angers.inra.fr/gddh13/), *Arabidopsis* (http://www.arabidopsis.org/), rice (http://www.ricedata.cn/gene/), and tomato (https://solgenomics.net/), respectively.
